# Trends in gastroesophageal reflux disease research: A bibliometric and visualized study

**DOI:** 10.3389/fmed.2022.994534

**Published:** 2022-09-29

**Authors:** Tai Zhang, Beihua Zhang, Wende Tian, Yuchen Wei, Fengyun Wang, Xiaolan Yin, Xiuxiu Wei, Jiali Liu, Xudong Tang

**Affiliations:** ^1^Xiyuan Hospital, China Academy of Chinese Medical Sciences, Beijing, China; ^2^Department of Gastroenterology, Xiyuan Hospital, China Academy of Traditional Chinese Medical Sciences, Beijing, China; ^3^Traditional Chinese Medicine Research Institute of Spleen and Stomach Diseases, Xiyuan Hospital, China Academy of Chinese Medical Sciences, Beijing, China; ^4^National Clinical Research Center for Chinese Medicine Cardiology, Xiyuan Hospital, China Academy of Chinese Medical Sciences, Beijing, China

**Keywords:** gastroesophageal reflux disease, VOSviewer, CiteSpace, bibliometrics, trends

## Abstract

**Background:**

Gastroesophageal reflux disease (GERD), a disorder resulting from the retrograde flow of gastric contents into the esophagus, affects an estimated 10–30% of the Western population, which is characterized by multifactorial pathogenesis. Over the past few decades, there have been many aspects of uncertainty regarding GERD leading to an ongoing interest in the field as reflected by a large number of publications, whose heterogeneity and variable quality may present a challenge for researchers to measure their scientific impact, identify scientific collaborations, and to grasp actively researched themes in the GERD field. Accordingly, we aim to evaluate the knowledge structure, evolution of research themes, and emerging topics of GERD research between 2012 and 2022 with the help of bibliometric approaches.

**Methods:**

The literature focusing on GERD from 2012 to 2022 was retrieved from the Science Citation Index Expanded of the Web of Science Core Collection. The overall publication performance, the most prolific countries or regions, authors, journals and resources-, knowledge- and intellectual-networking, as well as the co-citation analysis of references and keywords, were analyzed through Microsoft Office Excel 2019, CiteSpace, and VOSviewer.

**Results:**

A total of 8,964 publications were included in the study. The USA published the most articles (3,204, 35.74%). Mayo Clin ranked first in the number of articles published (201, 2.24%). EDOARDO SAVARINO was the most productive author (86, 0.96%). The most productive journal in this field was *SURGICAL ENDOSCOPY AND OTHER INTERVENTIONAL TECHNIQUES* (304, 3.39%). *AMERICAN JOURNAL OF GASTROENTEROLOGY* had the most co-citations (4,953, 3.30%). Keywords with the ongoing strong citation bursts were transoral incision less fundoplication, eosinophilic esophagitis, baseline impedance, and functional heartburn.

**Conclusion:**

For the first time, we obtained deep insights into GERD research through bibliometric analysis. Findings in this study will be helpful for scholars seeking to understand essential information in this field and identify research frontiers.

## Introduction

Gastroesophageal reflux disease (GERD) is a chronic, progressive, and relapsing condition, affecting up to 33% of the population worldwide (1, 2). It is estimated that up to 40% of the US population report GERD symptoms monthly and 20% weekly (3). GERD can be divided classically into non-erosive esophageal reflux disease (NERD) and erosive esophagitis (EE) (3). GERD occurs due to abnormal reflux of gastric contents into the esophagus, provoking symptoms or complications (4). Manifestations of GERD include esophageal syndrome consisting of heartburn, regurgitation, and reflux chest pain syndrome, or non-cardiac chest pain, and potentially, extra-esophageal syndrome (5). Esophageal exposure to gastric acid can be severe enough to cause ulcer or peptic stricture, Barrett’s esophagus (BE), and even esophageal adenocarcinoma (EAC) (6, 7). The management of GERD is complex as the clinical presentation is highly heterogeneous, and the pathophysiology involves an interplay of chemical, mechanical, psychologic, and neurologic mechanisms (8).

Although proton pump inhibitors (PPIs) are considered the mainstay of therapy for GERD and its complications, up to 40% of patients with NERD remain symptomatic while on standard therapy, and approximately 10–15% of patients with EE fail to achieve complete healing after 8 weeks of treatment (9–11).

In addition, GERD brings a heavy financial burden, negatively affects the quality of life, and reduces work productivity, especially in patients with severe and frequent symptoms (12–14). In the US alone, the financial burden is $9 to $10 billion per year in direct costs, mainly related to the use of PPIs as the first-line medication (2). GERD is also associated with 8,863,568 physician visits, 65,634 hospitalizations, and an estimated $12.3 billion in upper endoscopies in a year (15).

Despite decades of scientific work, there remain many unanswered aspects related to GERD, contributing to considerable attention from scholars. Hence, the disorder has been a subject of continued research interest, with a large body of literature published per year concerning epidemiology, etiology, pathophysiology, and therapeutics.

However, a mass of literature also represents great challenges for a researcher to identify the quality of the scientific publications, measure their scientific impact, or evaluate the research activity and scientific importance of countries, institutions, and scholars as well as gauge their scientific collaborations.

In this context, bibliometric approaches and visualization techniques are used to assess and visualize the qualitative, quantitative, and chronological aspects related to distinct areas of research (16).

The goal of the present study is to display the current status, knowledge components, research trends, and emerging topics of GERD research during the last decade by using knowledge maps. This article provides an overview of the studies and the scholarly contributions involved in this field and identifies the new developments.

## Materials and methods

### Source of the data and search strategy

Data were obtained from the Science Citation Index Expanded of the Web of Science Core Collection (WOSCC) of Clarivate Analytics on a single day, February 20, 2022. The full search strategy has been presented in Supplementary material. Articles or reviews which were published in English from 2012 through 2022 were included. The bibliographic records of the retrieved publications, including titles, abstracts, authors, affiliations, keywords, source, publication year, and cited references were downloaded in plain text and imported into CiteSpace and VOSviewer for analysis.

### Data analysis

CiteSpace (17), a freely available software developed by Professor Chaomei Chen from Drexel University in the USA, enjoys great popularity for visualizing the knowledge structure, distribution, and evolution of a given field. VOSviewer (18), another bibliometric software developed by Professor Nees Jan van Eck and Ludo Waltman from Leiden University in the Netherlands, has text mining capabilities to extract key elements from a large pool of scientific publications for constructing and visualizing co-authorship, co-citation, and co-occurrence network. Detailed definitions of each indicator, calculated in this study using CiteSpace and VOSviewer software are available elsewhere (19–23).

In the present study, CiteSpace was to (1) visualize collaborations among countries, institutions, and authors using knowledge maps; (2) perform a co-citation analysis of references; (3) generate the keyword co-occurrence cluster map; (4) depict the timeline view of co-occurring keywords; and (5) determine references and keywords with strong citation bursts. VOSviewer was applied to construct a keyword co-occurrence network. Microsoft Excel was used to demonstrate the temporal trends of publications.

## Results

### Publication output

The output of research related to GERD per year from 2012 through 2021 reached a stable range, resulting in a total of 8,964 documents, including 7,550 (84.23%) articles and 1,414 (15.77%) reviews.

**FIGURE 1:**
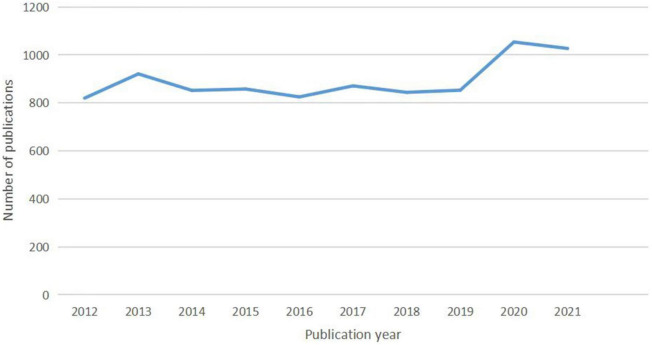
The number of articles published annually in GERD research.

The number of literature annually since 2012 is shown in Figure 1. The overall trend of publications roughly fell into two stages. In the first phase from 2012 to 2019, the annual number of articles ranging from 820 to 921, resulting in a total of 6,844 documents. In the second stage between 2019 and 2021, the annual publication output was in a period of rapid growth, with a total of 2,081 publications.

### Countries or regions and institutions analysis

GERD-related documents were published by 529 institutions from 138 countries or regions. The top 10 countries or regions and institutions are shown in Table 1 according to the publication number and the betweenness centrality. The USA was the leading producer of publications with 3,204 (35.74%) documents. Concerning productive institutions, Mayo Clin was the leader in publishing documents at 201 (2.24%).

**TABLE 1:** The top 10 countries or regions and institutions involved in GERD research.

Rank	Country	Centrality	Count (% of 8,964)	Rank	Institutions	Centrality	Count (% of 8,964)
1	The USA	0.08	3,204 (35.74)	1	Mayo Clin (the USA)	0.07	201 (2.24)
2	China	0.05	807 (9.00)	2	Northwestern Univ (the USA)	0.1	175 (1.95)
3	Japan	0.02	801 (8.94)	3	Univ Padua (Italy)	0.12	135 (1.51)
4	Italy	0.02	785 (8.76)	4	Univ N Carolina (the USA)	0.08	129 (1.44)
5	England	0.24	696 (7.76)	5	Karolinska Inst (Sweden)	0.04	109 (1.22)
6	Australia	0.03	398 (4.44)	6	Univ Milan (Italy)	0.05	105 (1.17)
7	South Korea	0.04	380 (4.24)	7	Univ Washington (the USA)	0.16	102 (1.14)
8	France	0.2	348 (3.88)	8	Baylor Coll Med (the USA)	0.04	100 (1.12)
9	Germany	0.11	331 (3.69)	9	Kings Coll London (England)	0.04	97 (1.08)
10	Canada	0.17	311 (3.47)	10	Univ Michigan (the USA)	0.02	94 (1.05)

**FIGURE 2:**
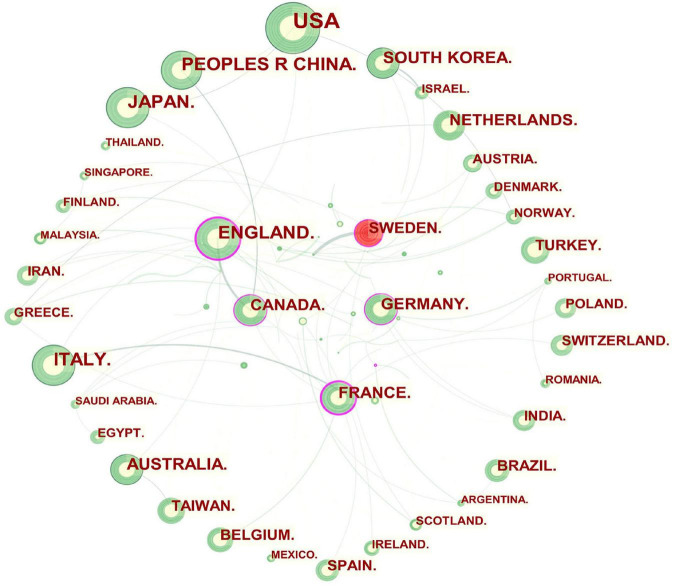
Network of countries and regions engaged in GERD research. In the network map, a node represents a country or region. The larger the area of the node is, the larger the number of publications. The thicker the curved line connecting nodes indicates the frequency with which they co-occur, as they indicate collaborative relationships. An isolated node without any connection is devoid of all collaboration. A node with a high betweenness centrality links two or more large groups of nodes. A node with a high betweenness centrality score exerts a strong influence on the network. A purple trim indicates a high degree of betweenness centrality. Red tree rings indicate bursts of citation. The greater the thickness of the red tree rings, the greater the bursts for the corresponding node.

In general, an article with authors from more than one country or institution denotes possible scientific partnerships (24). The visualization map of cooperation among countries or regions is presented in Figure 2. England, France, Canada, Germany, and Sweden, which were highlighted with purple rims had extensive ties and played a vital role in each of their partnerships.

**FIGURE 3:**
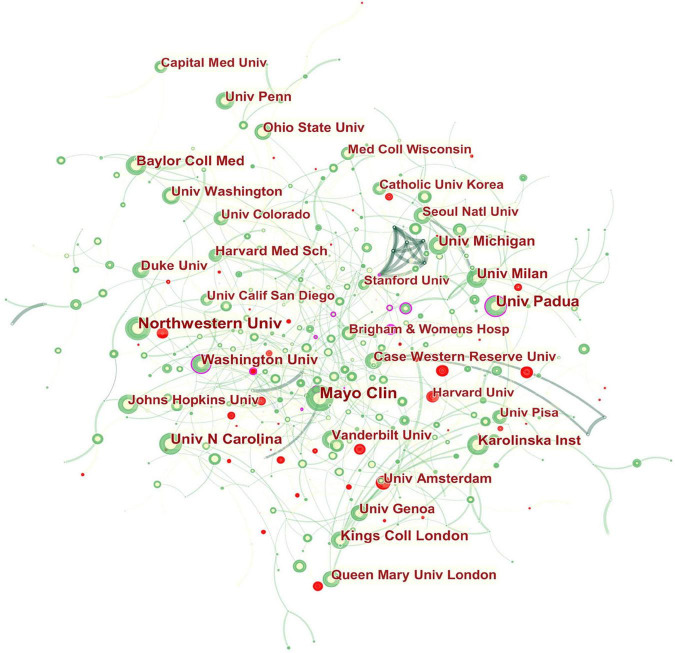
Network of institutions engaged in GERD research. In the network map, a node represents an institution. The volume of each node (institution) corresponds to the number of publications, and connecting lines between nodes indicate bidirectional relationships between the institutions; the thickness of the line indicates the strength of the bidirectional collaborative relationships. Isolated institutions lack any collaboration. A node with a high betweenness centrality links two or more large groups of nodes. A node with a high betweenness centrality score exerts a strong influence on the network. A purple trim indicates a high betweenness centrality. Red tree rings indicate bursts of citation. The greater the thickness of the red tree rings, the greater the bursts for the corresponding node.

The collaboration network of institutions is shown in Figure 3. The landmark nodes included Univ Padua and Univ Washington. They might be seen as the linking bridge between North American and European groups of collaborators, respectively.

### Authors

A total of 452 authors were included in the GERD studies. The top 10 active researchers are presented in Table 2. Among them, researchers from institutions in the USA authored most of the GERD literature. EDOARDO SAVARINO occupied the first position with 86 (0.96%) documents.

**TABLE 2:** The top 10 authors in GERD research.

Rank	Author	Count (% of 8,964)	Centrality
1	EDOARDO SAVARINO (Italy)	86 (0.96)	0.03
2	DANIEL SIFRIM (England)	55 (0.61)	0.1
3	MARCO G PATTI (the USA)	54 (0.60)	0.04
4	C PRAKASH GYAWALI (the USA)	53 (0.59)	0.22
4	JESPER LAGERGREN (Sweden)	53 (0.59)	0.05
5	NICHOLAS J SHAHEEN (the USA)	52 (0.58)	0.19
5	JOHN E PANDOLFINO (the USA)	52 (0.58)	0.1
6	YOSHIKAZU KINOSHITA (Japan)	50 (0.56)	0.07
7	RONNIE FASS (the USA)	49 (0.55)	0.03
7	VINCENZO SAVARINO (Italy)	49 (0.55)	0.03
8	MICHAEL F VAEZI (the USA)	44 (0.49)	0.06
8	PETER J KAHRILAS (the USA)	44 (0.49)	0.19
9	NICOLA DE BORTOLI (Italy)	42 (0.47)	0.02
10	HIROTO MIWA (Japan)	38 (0.42)	0.04

**FIGURE 4:**
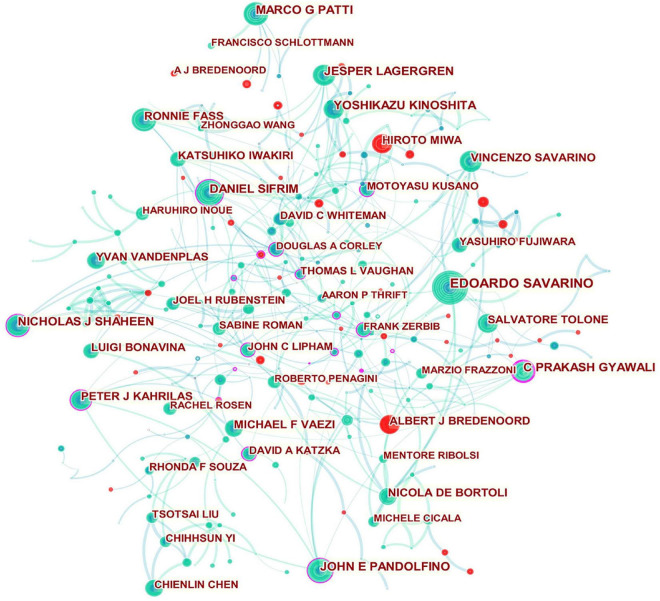
Network of authors in GERD research. In the network map, a node represents an author. The volume of each node (author) corresponds to the number of publications, and connecting lines between nodes indicate bidirectional relationships between the authors; the thickness of the line indicates the strength of the bidirectional collaborative relationships. Isolated authors lack any collaboration. A node with a high betweenness centrality links two or more large groups of nodes. A node with a high betweenness centrality score exerts a strong influence on the network. A purple trim indicates a high betweenness centrality. Red tree rings indicate bursts of citation. The greater the thickness of the red tree rings, the greater the bursts for the corresponding node.

According to Figure 4, the networks of partnerships are centered on DANIEL SIFRIM, C PRAKASH GYAWALI, NICHOLAS J SHAHEEN, JOHN E PANDOLFINO, PETER J KAHRILAS, MOTOYASU KUSANO, JOHN C LIPHAM, DAVID A KATZKA, THOMAS L VAUGHAN, DOUGLAS A CORLEY, and FRANK ZERBIB, suggesting they are most likely to initiate collaborative relationships and provide support for funding and resources in their respective communities.

### Journals and co-cited academic journals

There were 1,524 academic journals publishing articles related to GERD research. The top 10 journal outlets with high publication output are shown in Supplementary Table 1. Of these, *SURGICAL ENDOSCOPY AND OTHER INTERVENTIONAL TECHNIQUES* had the highest output (304, 3.39%).

In 1973, co-citation in the scientific literature was proposed by Marshakova I and Small H, which refers to the relationship between two documents that appear simultaneously in the bibliography of a third citing document (25, 26). Journal co-citation refers to the frequency with which any documents of two journals are cited together by the documents of other journals (27). A total of 1,244 co-cited scholarly journals published articles on GERD research. As shown in Supplementary Table 1, *AMERICAN JOURNAL OF GASTROENTEROLOGY* had the most co-citations (4,953, 3.30%).

### Co-cited references and references with citation bursts

Supplementary Table 2 presents the top 10 co-cited articles. These documents which were viewed as the knowledge base of GERD research deal primarily with the epidemiology of GERD, guidelines and consensus on the diagnosis and treatment of GERD, classification of esophageal motility diseases, and differential diagnoses for GERD [e.g., the functional esophageal disorders and eosinophilic esophagitis (EoE)].

As shown in Supplementary Table 3, the top co-cited references with the highest betweenness centrality were published from 2014 to 2020. They are considered to be key components in the intellectual base.

Burst detection is applied to detect publications or keywords that had a surge of their occurrences or citations, thus allowing for identifying subjects that have received intensive attention during a specific period (28). A citation burst has two attributes: strength and duration (29). As shown in Supplementary Figure 1, 25 references with the strongest citation bursts reflected research topics that received much attention over time. The document entitled “*Modern diagnosis of GERD: the Lyon Consensus*” written by Gyawali et al. (30) has received the strongest burst. Eleven articles (2, 30–39) still hold busts and their topics are considered research fronts in the field of GERD.

### Keywords analysis

#### Keyword co-occurrence

Essentially, a keyword describes the subject of a particular document succinctly and accurately and is used for indexing and cataloging purposes (40). Another prevalent way to track research topics in bibliometrics is keyword co-occurrence analysis (41). Keyword co-occurrence is based on the relevance of keywords which is determined by the number of documents in which they occur together. That is, when two keywords that reflect the research theme of an article appear in the same document, it is considered that there exist co-occurring relationships between the two terms. The higher the number of co-occurrences of two terms, the closer their relationship is.

**FIGURE 5:**
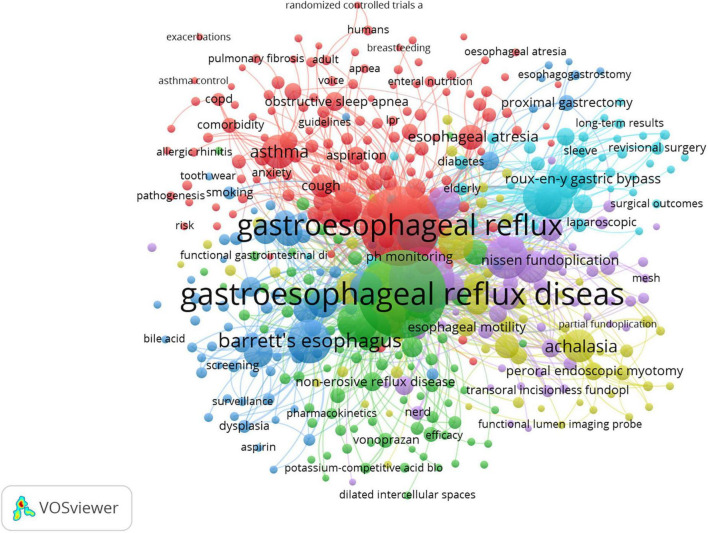
Map of keyword clustering with a minimum of 10 occurrences in GERD research. Minimum number of occurrence of a keyword = 10, minimum links strength = 10. There are 6 clusters of keywords.

A map of keyword co-occurrence is generated based on the frequency of occurrence of paired keywords. The visualization map of keyword co-occurrence by VOSviewer is shown in Figure 5. Each bubble represents a keyword. Bubble size indicates the frequency of occurrence of a keyword in publications, while the color of the bubble specifies the cluster to which it belongs. There were six clusters of keywords. Each cluster represents a distinct area of GERD research.

**Cluster 1 (Yellow):** The cluster demonstrated conditions mimicking GERD, particularly in regard to achalasia. Achalasia is considered the most studied and best-described primary motility disorder of the esophagus, characterized by abnormal relaxation of the lower esophageal sphincter (LES) and the absence of peristalsis. Swallowing rather than peristalsis causes simultaneous pressure waves in the esophagus (pressurization) that result in esophageal emptying in patients with this disorder, though the emptying is incomplete in these patients. In cases of advanced achalasia (types 1 and 2), the myenteric ganglia are fully replaced by fibrous tissue. Type 3 (spastic) achalasia is characterized by ganglia surrounded by chronic inflammatory cells, T (primarily), and B lymphocytes.

Patients with achalasia commonly complain of heartburn which may be mistaken for GERD (42). The cause of heartburn in achalasia is not fully understood. Perhaps the sensation occurs due to abnormal esophageal motor activity, or perhaps acid refluxes into the esophagus when the LES finally relaxes. It has been observed that patients with achalasia exhibit abnormal esophageal acid exposure, although it is still unclear whether the acid is hydrochloric acid refluxed from the stomach or lactic acid produced by carbohydrate fermentation in the esophagus after retention (43). Achalasia patients may experience heartburn resistant to PPI treatment, a condition which has occasionally led to Nissen fundoplications being performed to treat heartburn misattributed to GERD. In this context, Nissen fundoplication can result in debilitating dysphagia (44).

It is estimated that achalasia spectrum disorders are identified in 1–2.5% of patients who undergo esophageal manometry prior to anti-reflux surgery (45), and this test should be considered if esophageal symptoms fail to improve after acid suppressive therapy. To prevent this potentially catastrophic mistake, a preoperative esophageal manometry is recommended for patients considering fundoplication as a treatment for GERD (46).

**Cluster 2 (Purple):** This cluster is centered on anti-reflux surgery. The first fundoplication for EE was performed by Dr. Rudolf Nissen in 1955, which was then published in 1956 (47, 48). Following an initial period of success and widespread adoption, this revolutionary procedure has begun to experience significant declines owing primarily to high complications (48, 49). With the introduction of PPIs in the 1980s, patients who did not receive adequate symptomatic relief or had advanced esophagitis despite optimal medical treatment were only considered for traditional open surgery (49, 50). In 1991, Dallemagne et al. (50) reported their first experience with a laparoscopic Nissen fundoplication (LNF) and noted symptomatic improvement and no mortality from the procedure. The laparoscopic anti-reflux surgery (LARS), which includes LNF and partial wraps, has resulted in significantly less morbidity and mortality and a shorter recovery period than the open approach (51). It has since become the preferred procedure among patients with complications resulting from GERD. Additionally, this may be a more cost-effective procedure for those in the younger age group.

Despite the initial report by Dallemagne et al. (50) indicating no mortality, it is notable that there are serious adverse events, which may include mortality. In the LOTUS trial, which is a randomized, open, parallel-group trial conducted in Europe, it was noted that, at 5 years, although heartburn and regurgitation were better controlled in the LARS group, a higher prevalence of dysphagia, bloating and flatulence were observed in the LARS group than those in the esomeprazole group (52). In addition, there was a 3% in-hospital mortality rate and a 26.8% rate of serious adverse events in the LARS group (52). Consequently, laparoscopic surgery is underutilized due to its perceived side effects, generally indicated for patients with long-standing severe diseases and large hiatal hernias. In light of such experience and results, further investigation into procedural strategies that minimize post-operative side effects while maximizing LARS therapeutic benefits continues.

The results of a landmark prospective study by Spechler SJ et al. (53) demonstrated the benefit of surgical treatment in patients with reflux hypersensitivity (RH). LNF or medical therapy was administered to patients with refractory heartburn who had RH or pathological acid reflux on esophageal function testing. Reflux surgery proved superior to medical therapy; 71% of patients with RH had success, compared to 62% of patients with abnormal acid reflux (53). This study demonstrates that RH considered a functional esophageal disorder until recently, responds to fundoplication. Yet one-third of the patients in the surgical arm did not respond, and serious risks of fundoplication need to be carefully evaluated against the benefits for each patient.

**Cluster 3 (Light blue):** With its emphasis on laparoscopic sleeve gastrectomy (LSG) and laparoscopic Roux-en-Y gastric bypass (LRYGB), this cluster has primarily focused on bariatric surgery. Despite the fact that weight loss after bariatric surgery improves GERD symptoms, the degree of symptom relief varies according to the type of procedure. There was evidence to suggest that patients undergoing LRYGB, adjustable gastric band, and LSG all showed significant relief in GERD symptom scores after 6 months of follow-up (54). A maximum GERD score improvement occurred in patients with LRYGB, followed by an adjustable gastric band and LSG (54). Prachand et al. (55) reported that GERD resolution was still higher after RYGB (76.9%) compared to biliopancreatic diversion and duodenal switch (48.6%) in the super-obese patients, even though the latter procedure offered better weight loss, better control of diabetes mellitus, hypertension, and hyperlipidemia.

The Swiss Multicenter Bypass or Sleeve Study (SM-BOSS) reported that GERD remission was greater after RYGB, at 60.4% than LSG, at 25% after 5 years (34). Further, GERD symptoms worsened in 31.8% of patients with LSG compared to 6.3% with RYGB (34). *De novo* GERD developed in 31.6% of LSG patients after 5 years, *versus* 10.7% of patients with RYGB (34). The results indicated that GERD symptoms were more prevalent after LSG compared to RYGB.

Because of the LSG’s robust weight loss effect and simple surgical procedure, it has grown increasingly popular among the bariatric community (56). However, the development of *de novo* GERD following LSG has recently gained attention, which is estimated to occur in 73% of patients (57, 58). Compared to other bariatric procedures like LRYGB, LSG has been estimated to increase the risk of developing *de novo* GERD by five times (59). An increased risk of GERD can also result in adverse consequences, such as BE and perhaps even adenocarcinoma (60, 61). Some mechanisms are proposed to account for *de novo* GERD development, such as altered anatomy of the gastroesophageal junction, disruption of the sling fibers, altered LES function, narrowing of the stomach, dilation of the fundus, increased intragastric pressure, and the concomitant presence of a hiatal hernia (62–65).

**Cluster 4 (Dark blue):** This cluster mainly pertains to BE. In response to gastroesophageal reflux (GER), BE develops from an acquired metaplastic epithelial change in the esophagus. Based on a systematic review and meta-analysis of 19 studies involving 43,017 subjects, GER symptoms are significantly associated with a higher risk of endoscopically suspected and histologically confirmed BE (66). This risk increases further when patients exhibit weekly symptoms (66).

In fact, up to half of the patients with BE do not complain of reflux symptoms, although the magnitude of the risk for BE and EAC has been reported to be higher in patients with prolonged symptoms of GER and a younger age of reflux onset (67, 68). Additionally, symptom relief is a poor indicator of acid control, with pH normalization seen in 85% of patients on PPIs (67). There has been evidence that acid reflux can cause inflammation in the distal esophagus via activation of cyclooxygenase-2 (COX-2), c-myc, and mitogen-activated protein kinase (69, 70). *Ex vivo* studies have demonstrated the over-expression of COX-2 in Barrett’s epithelium and in the EAC (69, 70).

In addition, recent research has shown indirect effects of acid on the epithelium of the esophagus. Some pro-inflammatory mediators have been implicated in animal and human models, such as interleukin (IL)-8, platelet-activating factor, and interferon-γ (71–73). In response to the release of these mediators, immune cells are recruited to the esophageal mucosa, leading to a cascade of inflammatory pathways that produce reactive oxygen species (ROS) and further cell injury (71–73). Research has demonstrated that acidic media induces pro-proliferative and anti-apoptotic effects in esophageal cells (74).

It has been proposed that bile reflux or duodenogastric reflux also contributes to BE with bile acids in the refluxed material of patients with BE. Bile acids’ effects on the mucosa of the esophagus have been linked to both cytotoxic mechanisms and the activation of proto-oncogene and c-myc, which contribute to inflammation-related carcinogenesis (75). Bile acids, as detergent molecules, have the potential to solubilize cell membranes, but their ability to penetrate cell membranes depends on being neutralized and therefore lipophilic. When exposed to acidic pH, bile acids become non-ionized, enter cells, and cause mucosal injury and inflammation (67, 76). The conjugation of bile acid with gastric acid increases the viability of acid hydrolase, destroys lysosomal membranes, and leads to reverse hydrogen ion diffusion (77). Additionally, previous studies have suggested that acid and duodenogastric reflux are synergistic, with the latter contributing to mucosal damage rather than reflux symptoms (77).

Bile acids may trigger the release of inflammatory mediators, which may contribute to the development of BE, as well as carcinogenesis in patients with BE. This may involve an increase in levels of IL-6, IL-8, COX-2, and tumor necrosis factor-α, along with the recruitment of inflammatory cells (78). The increased pro-inflammatory cytokines and cells were not observed in an acid-only cohort, indicating that bile acids directly contribute to esophageal damage (78). Moreover, there is evidence that bile acids, when present in an acidic environment, may induce the release of ROS that can, in turn, cause oxidative stress, which leads to DNA damage, and increases the risk of cellular metaplasia (79, 80).

The esophagus is bathed in up to 3 mg/ml pepsin (up to 73 times per day) during chronic GERD that persists for years. A study by Samuels et al. (81) established the expression of pepsinogen mRNA in BE mucosa; acute non-acid pepsin, on the other hand, was able to induce pro-inflammatory mechanisms and cell migration in esophageal cells *in vitro*. Furthermore, mitochondrial cristae degeneration and mitochondrial dysfunction are also associated with non-acid pepsin-mediated injuries and may be caused by the suppression of B cell lymphoma-2 family proteins involved in the maintenance of mitochondrial structure and homeostasis (82–85). Mitochondrial dysfunction is characterized by ROS causing mitochondrial DNA damage, a common event during chronic inflammation, and is associated with the metaplastic and preneoplastic responses including BE (86, 87). As reported in a study by Samuels et al. (88), chronic (2–4 weeks) exposure to pepsin induced the production of IL-8, a neutrophil chemoattractant and a trigger for cellular proliferation and angiogenesis and, therefore, facilitates inflammation-mediated tumor initiation. In patients with GERD, IL-8 levels are elevated and are highest in patients with BE and EAC; following anti-reflux surgery, IL-8 levels are reduced in patients with BE (89). Chronic pepsin treatment also results in a shift from the normal esophageal cytokeratin profile of *KRT10* high and *KRT8* low to the BE-type cytokeratin profile of *KRT10* low and *KRT8* high (88, 90).

Pepsin is erosive to esophageal tissue when it is present in acid reflux. When weak and non-acidic reflux occurs, as seen in patients taking PPIs, the enzyme activity of pepsin is temporarily inhibited, allowing interaction with an unidentified receptor, endocytosis, and retention in acidic intracellular vesicles where its activity can be restored (82, 83). Mechanistically, in BE, there are diverse cell types including the oxyntocardiac mucosa, which contains parietal cells that secrete gastric acid, and chief cells that secrete digestive enzymes (91). Oxyntocardiac mucosa is a precursor to intestinal metaplasia and EAC (92) and often occurs in the context of EAC (93). Further, in GERD-associated metaplasia and dysplasia, pepsinogen and proton pump mRNA were exclusively expressed; no expression was observed in the non-cancerous esophagus and squamous cell carcinomas are not associated with GERD (88). In this manner, there is a hypothesis that the refluxed or locally produced acid may facilitate the conversion of locally synthesized pepsinogen to active pepsin and reactivate intracellularly deposited refluxed pepsin, which might account for the association of GERD with metaplastic changes and neoplastic lesions (88).

**Cluster 5 (Red cluster):** With “asthma,” “cough,” and “obstructive sleep apnea-hypopnea syndrome,” GERD-induced respiratory disease is the focus of this cluster. Taking asthma as an example, its frequent coexistence with GERD raises the possibility that these diseases may have shared mechanisms.

The prevalence of asthma among GERD populations has been compared in many studies, with results showing a modest or no correlation (3, 94, 95). As reported in a systematic review involving 28 studies, the average prevalence of asthma among patients with GERD was 4.6% (compared to 3.9% among controls), with an overall ratio of 2.3 [95% confidence interval (CI): 1.8–2.8] (96). Even if the evidence indicates an increased prevalence of asthma among patients with GERD, most studies were cross-sectional or case-control studies; therefore, the causal direction of the association could not be determined (96).

Studies have also examined the relationship between GERD and asthma severity and exacerbation; GERD is clearly associated with asthma exacerbations. For example, a significant correlation has been reported between the presence of reflux disease and an increase in exacerbations [odds ratio (OR) = 4.9; 95% CI: 1.4–17.8] and hospitalizations in asthmatic patients (97, 98). Further evidence for this association was presented in a recent meta-analysis involving 32 studies of 1,612,361 patients of all ages (99). The study found that GERD was associated with asthma exacerbations (OR = 1.27; 95% CI: 1.18–1.35) and exacerbations requiring oral corticosteroid therapy (OR = 1.24; 95% CI: 1.09–1.41) (99).

There are two main mechanisms involved in GERD associated with asthma severity and exacerbations, which include (1) aspiration of gastric contents directly affecting pulmonary parenchyma and aspiration of contents up to the pharynx causing the symptoms by stimulating irritant receptors (100, 101) and (2) gastric contents reflux into the lower esophagus, which may cause symptoms by increasing bronchial reactivity, or by causing bronchoconstriction as a result of the vagal nerve stimulation (102, 103). Furthermore, asthmatics often experience hyperinflation, and with hyperinflation, there is an increase in intrathoracic pressure as a result of an increased lung capacity, which may induce descent of the diaphragm in this setting, increasing the gradient between the abdomen and the chest, leading to herniation of the LES into the chest and thus affecting LES tone (104). Bronchodilators used for asthma can also relax the LES, causing gastric contents to reflux. Therefore, asthma and GERD seem to be closely related physiologically, as asthma may cause GER and GERD may aggravate asthma symptoms, although the causal relationships are not well understood and demonstrated.

**Cluster 6 (Green cluster): T** his cluster focused on potassium-competitive acid blockers (P-CAB) in GERD, particularly in relation to NERD, the most common type.

Currently, P-CABs are primarily approved in Asia, and they were thought to be an attractive option for patients with NERD due to their quick onset of action on gastric acid secretion. Vonoprazan (TAK-438) is the most extensively studied of this new class of acid suppressants, which has been available to the Japanese market since 2015 (105). According to the first randomized, double-blind, placebo-controlled, multicenter study conducted in patients with NERD with no mucosal changes (Grade N) or minimal mucosal changes, such as mucosal redness, or turbidity (Grade M) and recurrent acid reflux symptoms, the number of heartburn-free days with vonoprazan (10 or 20 mg daily) was not superior to placebo, even though it was found that both doses of vonoprazan significantly decreased the mean severity of heartburn (106). As the results of the first trial were close to showing a significant difference, another phase III trial using vonoprazan 10 mg in NERD patients was conducted (107). The proportion of days without heartburn was not significantly different between the vonoprazan and placebo groups among patients with NERD in the full analysis (107). In the per-protocol-set analysis, however, the complete heartburn resolution in the fourth week of treatment was significantly higher in the vonoprazan group compared with the placebo group (*p* = 0.0023) (107). Therefore, it remains surprising that vonoprazan failed to achieve superiority in the primary efficacy outcome compared with placebo and therefore failed to receive approval for the NERD indication.

These low response rates suggest that other factors are responsible for symptom generation in this group of patients. The problem has been noted because NERD patients have been indicated as having inferior treatment outcomes with PPIs compared with EE patients (108). The partial response seen with vonoprazan monotherapy can be attributed to weakly acidic reflux or functional heartburn (FH), according to Kawami N et al. (109) and Abe et al. (110).

In spite of these results, the drug appears to be effective in the treatment of PPI-resistant NERD. A small retrospective study found that 69.2% of PPI-resistant GERD patients reported an improvement in self-reported GER symptoms and quality of life measured by GERD-Q score following their medication from a PPI to vonoprazan (111). Similarly, vonoprazan was reported to be effective in relieving the GER symptoms in patients with PPI-resistant NERD by Shinozaki et al. (112). Accordingly, these studies do not eliminate the possibility that P-CAB might be useful in NERD; however, larger randomized controlled trials are required to confirm these findings.

Tegoprazan (CJ-12420) is a benzimidazole derivative, approved in 2018 for treating gastric ulcers and GERD in South Korea. In South Korea, tegoprazan has been approved as the first P-CAB for NERD. According to the study by Kim et al. (113), which assessed complete relief of heartburn and regurgitation in patients with NERD following 4-week tegoprazan therapy; 42.5, 48.5, and 24.2% of patients had completely resolved major symptoms receiving tegoprazan 50 mg, tegoprazan 100 mg, and placebo, respectively. Both tegoprazan groups experienced higher rates of complete heartburn resolution and heartburn-free days than the placebo group (*p* < 0.05 for all) (113). The authors concluded that tegoprazan relieved NERD symptoms in an effective and sustained manner, thus providing a viable therapeutic option for the treatment of NERD (113). In this study, patients with NERD were defined as those who frequently experienced heartburn and regurgitation and had a normal endoscopy results. However, the clinical relevance of this definition is still open to question today given its heterogeneity. Despite statistically significant improvement over placebo, there appears to be no clinically significant advantage over PPIs (114, 115), although no PPIs were compared with this drug. Therefore, unless negative endoscopy with positive 24-h-pH testing was used to define confirmed NERD, it remained unclear how much better tegoprazan and other PCABs may be at reducing GER symptoms. Furthermore, large-scale, prospective, randomized controlled studies using PPI as a comparator may further inform research on symptomatic control in patients with NERD.

**FIGURE 6:**
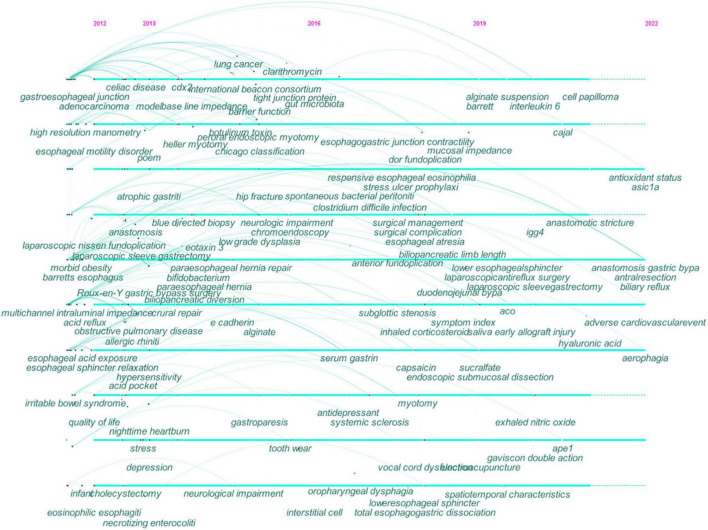
Timeline view of co-occurring keywords map in GERD research. The year placed at the top of the view corresponds to the earliest year when each keyword appeared. Each node represents a keyword. The links represent the co-occurrence of keywords and the colors represent the average year of publication for each node. Each cross corresponds to the bursts of keyword co-occurrence.

Figure 6 shows the timeline view of keyword co-occurrence, from which the evolution of research topics can be examined with time.

During the early years from 2012 to 2016, GERD research began to focus on (1) EoE, BE, EAC, and esophageal motility disorders; (2) morbid obesity, stress, and depression; (3) nighttime heartburn; (4) gastroparesis, celiac disease, and irritable bowel syndrome; (5) chronic obstructive pulmonary disease and allergic rhinitis, (6) high-resolution manometry, multichannel intraluminal impedance-pH monitoring, and baseline impedance; (7) botulinum toxin and *Bifidobacterium*; (8) alginate; (9) RYGB and LSG; (10) Heller myotomy, per-oral endoscopic myotomy, LNF, and para-esophageal hiatal hernia repair; (11) esophageal sphincter relaxation, esophageal hypersensitivity, and acid pocket; (12) caudal-type homeobox transcription factor 2, eotaxin-3, and E-cadherin.

From 2016 to 2019, research in this field focused on (1) hip fracture, *Clostridium difficile* infection, and spontaneous bacterial peritonitis; (2) systemic sclerosis; (3) subglottic stenosis; (4) antidepressant; (5) Toupet fundoplication; (6) capsaicin; (7) interstitial cell.

Recent research trends from 2019 to 2022 included (1) IgG4-related disease, aerophagia, and early allograft injury; (2) the symptom index and exhaled nitric oxide; (3) biliary reflux; (4) adverse cardiovascular event; (5) Gaviscon double action; (6) hyaluronic acid; (7) antioxidant status; (8) acid-sensing ion channel 1a; (9) apyrimidinic endonuclease I and IL-6;

#### Burstness of keywords

**FIGURE 7:**
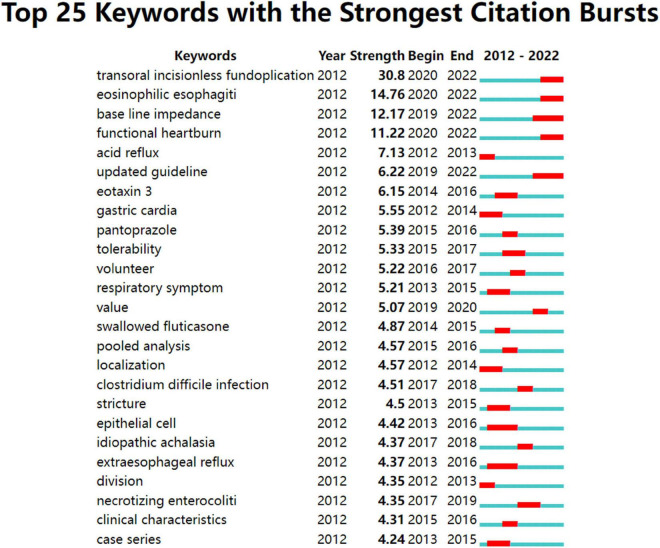
Top 25 keywords with strong citation bursts in GERD research. A blue bar represents the period from 2012 to 2022, whereas red line segments represent the time slices during which keyword bursts occur, i.e., rapid increases in citation counts.

The strength and duration of the 25 keywords with the strongest citation bursts are shown in Figure 7. The most intense one is “transoral incisionless fundoplication (TIF)” (30.8), followed by “EOE” (14.76), “baseline impedance” (12.71), and “functional heartburn” (11.22). In addition, these keywords had ongoing bursts, representing the research frontiers in this field.

## Discussion

### General information

As shown in Table 1 and Figure 2, Europe (Italy, England, France, and Germany), Asia (China, Japan, and South Korea), North America (the USA and Canada), and Oceania (Australia) were active in GERD research. The betweenness centrality of a node refers to the ability to connect to other parts of the network. A source with a high betweenness centrality is more likely to broker information flows, proving its diversity in collaboration and revolutionary potential (116). It is demonstrated that collaboration exerts a positive effect on research quality, with an increase in the number of coauthors correlating with citation impact, especially when international collaboration is involved (117, 118). Thus, England, France, Canada, Germany, and Sweden were pivotal in bridging global cooperation and influence in the GERD field.

As shown in Table 1 and Figure 3, high-yield institutions are concentrated in North America and Europe. Overall, the USA-based institutions had limited collaborative relationships globally. For example, the most prolific institution, Mayo Clinic, actively collaborated with Mahidol Univ, Northwestern Univ, Univ Newcastle, and AstraZeneca, indicating a lack of intra-continental cooperation. Northwestern Univ which ranked second in scientific output, frequently cooperated with Univ Calif San Diego, Texas A&M Univ, Mayo Clinic, Univ Colorado, and Lyon I Univ, whose collaboration, therefore, tended to be an intra-country phenomenon.

As shown in Table 2 and Figure 4, scholars from the USA and Italy stood out as the leading force. However, Asian researchers were scarce in the top ranking, especially Chinese and Korean ones, which was similar to the landscape of top institutions without any Asian institutions included. It is possible that GERD research in Asia was hampered by inadequate resource-, intellectual- and knowledge-sharing. Thus, Asian regions such as Japan, China, and South Korea are encouraged to pursue international scientific cooperation while increasing research productivity, which is closely linked to improved research quality and enhanced research capacity.

In addition, GERD research showed an overall trend of specialization, with most productive researchers interested in esophageal gastroenterology and upper gastrointestinal surgery. This was also reflected in the author’s collaboration network. For example, C PRAKASH GYAWALI, who had the highest centrality indicator, possessed strong research collaboration and held significant scientific impact in this domain, whose co-authorship community consisted of researchers with expertise in esophageal motility, esophageal pathology, and esophageal surgery, including FRANK ZERBIB (France), RONNIE FASS (the USA), SABINE ROMAN (France), BENJAMIN D ROGERS (the USA), CHRISTINA BROCK (Denmark), JOHN E PANDOLFINO (the USA), AMIT PATEL (the USA), and MICHAEL F VAEZI (the USA).

Based on Supplementary Table 1, studies on GERD have primarily been published in journals dealing with gastrointestinal motility, esophageal surgery, gastrointestinal endoscopy, pediatric gastroenterology, and metabolic and bariatric surgery, which is indicative of GERD being multifactorial and affecting almost all age groups.

The number of co-citations was generally applied to appraise the academic performance of a researched subject and its impact on the scientific community (119). Journals with high co-citations are referred to as mainstream journals in this field. There were primarily high co-citations found in journals with high IF and journals located in Q1, indicating that GERD research published in top-tier journals has consistently drawn attention.

Furthermore, there is a concurrence of *SURGICAL ENDOSCOPY AND OTHER INTERVENTIONAL TECHNIQUES*, *DISEASES OF THE ESOPHAGUS*, *WORLD JOURNAL OF GASTROENTEROLOGY*, and *DIGESTIVE DISEASES AND SCIENCES* in the top productive journals and highly co-cited ones, implying they were deemed core journals in the GERD field.

### Knowledge base

In addition to the conceptual structure using the keyword co-occurrences to identify clusters of research themes that extensive research has been dedicated to shown in Figure 5, we took a closer look at the top co-cited references (36, 60, 120–124) with the highest betweenness centrality listed in Supplementary Table 3 to shed light on the key components of the intellectual base of GERD research. The topics of these references were closely related to cluster 3 (bariatric surgery) and cluster 4 (BE) displayed in Figure 5. In addition, potential risks associated with PPI use in the long term are also of concern. These references with their key findings are summarized in Supplementary Table 4 to provide an overview.

### Research frontiers

Figure 7 shows keywords with continuing strong citation bursts that denoted research frontiers within this field. The emerging topics mostly focus on endoscopic treatments, such as TIF, as well as the differential diagnosis of GERD including EOE and FH, utilizing emerging modalities such as mucosal impedance.

### Endoscopic treatments

Even though many patients with GERD experience relief following dietary and medical management alone; however, a substantial percentage of these patients remain resistant to medical management or reject long-term pharmacotherapy, citing concerns regarding possible side effects. In terms of surgical treatment for GERD, the Nissen fundoplication is currently the gold standard and the mainstay of surgical management for GERD; however, the invasive nature of the procedure makes it less appealing to many patients, particularly those with less severe symptoms.

With regard to endoscopic treatments for GERD, they offer a true minimally invasive option with less pain and shorter hospital stays as well as the ability to alleviate post-operative dysphagia and the inability to vomit or belch. These mainly include radiofrequency therapy, TIF, and endoscopic suturing. As identified in our analysis, TIF has generated great interest for research; we came closer to this emerging approach.

There have been multiple non-comparative studies as well as randomized controlled trials that compare TIF with PPI controls (124–128). Based on a meta-analysis of 963 patients across 18 studies, the relative risk of response to TIF therapy compared with PPIs or sham treatment was 2.44 (95% CI: 1.25–4.79; *p* = 0.0009) (129). Even though the total number of reflux events decreased after TIF compared with either the PPIs or the sham group, the AET and the number of acid refluxes did not decrease significantly (129). The long-term follow-up further revealed that PPIs usage increased with time after the procedure, although at a lower dose (129).

Another meta-analysis was conducted using data from randomized studies evaluating TIF compared to sham or PPI therapy (130). This study aimed to evaluate the efficacy and long-term outcomes of TIF therapy in patients with refractory GERD who were on optimized PPI therapy (130). It was found that the TIF subjects at 3 years showed significantly improved esophageal pH, a decrease in PPI utilization, and improved quality of life (130). Recent studies have also demonstrated favorable long-term outcomes at 5 years, and preliminary results at 10 years (131–133).

With TIF, patients with GERD who have a hiatal hernia under 2 cm and a Hill grade of less than 3 can be safely and effectively treated. The American College of Gastroenterology’s 2021 Clinical Guideline for the Diagnosis and Management of GERD also recommends using TIF in patients with troublesome symptoms who refuse surgery or have a mild form of GERD, including those with hiatal hernias of less than 2 cm and esophagitis with a LA grade of A or B (134). Interestingly, TIF is being performed in combination with laparoscopic hiatal hernia repair to increase accessibility to TIF for patients with GERD and hiatal hernias larger than 2 cm (135).

#### Esophageal electrical impedance technologies

Despite the absence of macroscopic signs of injury, mucosal impedance reflects the degree of permeability of the mucosa and is correlated with its integrity, with low values indicative of an alteration in the intercellular spaces and tight junctions found in GERD (136–140).

There are several methods available for measuring esophageal mucosal impedance, including mean nocturnal baseline impedance (MNBI), high-resolution impedance manometry (HRIM)-derived measurements, and mucosal impedance probes (single-channel or balloon).

The MNBI may serve as a surrogate marker of pathological reflux, whereas the AET is subject to significant day-to-day variability (30). MNBI has been extensively studied in the field of reflux disease for its role in diagnosis and phenotyping.

The established cut-off value for MNBI was 2,292 Ω, and it has been shown to potentially distinguish GERD patients from those with FH (138, 141). The MNBI levels are proportionally higher, moving from EE to NERD to RH, whereas the values are normal in FH and healthy controls (138, 141). Further, MNBI results have been linked to medical and surgical outcomes. A low MNBI is shown to be an independent predictor of response to anti-reflux therapy even in patients with borderline AET (4-6%) (142, 143). The MNBI value improves with the healing of EE, making it an interesting adjunctive measure of acid control (144, 145). The MNBI values were also found to distinguish PPI-responsive from PPI-refractory patients with heartburn and normal conventional impedance-pH variables, with a normalization of the MNBI values following anti-reflux surgery (139, 146). However, Ribolsi et al. (147) failed to identify the difference in MNBI between PPI responders and non-responders in NERD patients and they concluded baseline impedance didn’t predict PPI response and has no association with reflux perception. To date, the role of MNBI in NERD patients who are PPI-refractory has not been thoroughly examined.

However, despite its increasing popularity as an assessment of mucosal integrity, MNBI requires overnight catheter placement and manual software manipulation. To simplify impedance recording further, it has been suggested that baseline impedance be measured during HRIM. Ravi et al. (148) compared baseline impedance obtained from HRIM with MNBI and AET among 29 GERD patients and 26 controls. Baseline impedance measurements were made during the 15 s landmark phase of HRIM (148). There was a good correlation between baseline impedance via HRIM and MNBI (*r* = 0.59, *p* < 0.01) and the low baseline impedance via HRIM had high diagnostic accuracy for GERD (148). The authors concluded that the HRIM baseline impedance provided the potential for accurate, cost-effective, and less-invasive diagnostics for GERD (148). Although HREMI has limited evidence for this purpose, available data regarding HRIM-derived impedance seem promising (148, 149).

In a pioneering effort, Vaezi MF and colleagues have developed a mucosal impedance catheter to ensure the impedance sensors onto the esophageal mucosa through the working channel of the gastroscope. An initial study was carried out using a single-channel mucosal impedance catheter that was inserted through the accessory channel of an endoscope to measure the direct mucosal impedance of 19 patients with EE, 23 with NERD, and 27 controls (150). GERD patients had a significantly lower distal esophageal impedance than non-GERD patients (150). The axis of the distal-proximal esophagus showed a significant and graded increase in mucosal impedance in GERD patients (150). Further, in 61 patients with EE, 81 patients with NERD, 18 patients with achalasia, 15 patients with EoE, and 93 controls, Ates et al. (151) evaluated the utility of mucosal impedance utilizing a single-channel probe through the endoscope. A significant decrease in mucosal impedance values was observed in patients with GERD and EoE when compared with non-GERD or achalasia patients (151). EoE also had a distinct mucosal impedance pattern compared with GERD, with low values observed along the length of the esophagus at 2, 5, and 10 cm above the squamocolumnar junction, rather than a clear distal to proximal mucosal impedance gradient (151).

Although the single-channel mucosal impedance catheter introduced in these studies as a proof of concept was attractive due to its simplicity, some were concerned that a point impedance measurement could result in significant inter-provider variability due to intraluminal air or liquid, catheter movement, and insufficient mucosal contact. Consequently, a novel balloon mucosal impedance catheter system composed of 10 cm axial columns of impedance sensors mounted on a balloon that is inflated to enable a 360-degree measurement of mucosal impedance across the tubular esophagus was developed in order to eliminate these concerns. Mucosal impedance patterns acquired by the balloon-mounted mucosal impedance catheter system have been proven safe and reliable for differentiating patients of GERD, EoE, and non-GERD (152). Despite the need for more data, the initial results from the mucosal impedance balloon catheter are promising; furthermore, its ease of data acquisition, simplicity of interpretation, and rapidity of the test make it appealing in comparison to MNBI interpretation which entails cumbersome ambulatory catheter monitoring and manual analysis of tracings (153).

During upper endoscopy, biopsies may be obtained to rule out structural abnormalities that may cause heartburn, such as EoE. However, the distribution of eosinophilia in EoE is segmental or patchy, requiring multiple biopsies for detection, and even aggressive biopsies may only cover a small section of the entire esophagus. Histologically, in addition to eosinophil infiltrates, one of the key histologic characteristics of EoE involves the appearance of dilated intercellular spaces (DIS) between epithelial cells, thus leading to esophageal epithelial permeability (154). Accordingly, mucosal impedance is a valuable method for measuring the conductivity of the esophageal epithelium and can reliably separate EoE from GERD and other non-GERD conditions.

Katzka et al. (155) evaluated 10 active and 10 inactive EoE patients and compared mucosal impedance measurements with biopsy results in a study using the single-channel mucosal impedance probe. An inverse correlation was reported between mucosal impedance measurements and eosinophil/high-power field and spongiosis on esophageal biopsies in EoE (155). Patients with active disease had a lower mucosal impedance, and a cut-off value of 2,300 Ω identified patients with active EoE with 90% sensitivity and 91% specificity, and high-grade DIS with 89% sensitivity and 82% specificity (155). Furthermore, this measurement was validated in the pediatric population, showing that patients with active EoE have lower mucosal impedance than those with inactive EoE, NERD, or controls (156). Another study by Alexander et al. (157) compared mucosal impedance using a balloon catheter and eosinophil counts in endoscopic biopsies in 10 controls, 18 patients with active EoE, and 5 patients with inactive EoE. In analyzing individual impedance measurements (18 per patient), it was found that control patients had normal mucosal impedance values 95.6% of the time as compared to 29.2% in EoE patients, and no EoE patient had uniformly normal mucosal impedance (157). However, a poor correlation was reported between peak esophageal eosinophil counts, EoE activity, and mucosal impedance (157).

Overall, mucosal impedance, measured with a variety of techniques, either ambulatory setting or single-channel and balloon-based, appears promising and may be able to differentiate GERD from EoE as well as track the histologic progression of EoE (151, 155–159). Currently, however, data regarding mucosal impedance measurements in EoE are limited. It appears that mucosal impedance, peak eosinophil counts, and EoE disease activity in individual patients do not correlate well, according to Alexander et al. (157); even with a balloon-probe mucosal impedance catheter system, mucosal impedance measurements do not assess the whole esophagus, which may not accurately evaluate the patchy epithelial change associated with EoE. Therefore, it is still necessary to obtain biopsies for EoE assessment and mucosal impedance cannot currently replace biopsy acquisition for assessing EoE disease activity (153).

PPI-resistant heartburn can be caused by functional heartburn, and approximately 50% of patients with refractory heartburn are attributed to functional heartburn (160, 161). Furthermore, about half of patients with NERD who fail to improve after treatment with P-CABs, in fact, have functional heartburn (109, 162). In a recent survey, FH was cited by healthcare providers as the most common reason for incomplete response to PPI therapy despite following dosing suggestions (163). It is, therefore, necessary to distinguish GERD, in particular NERD, from FH, as FH is not reflux-related and PPIs lack therapeutic value, except in cases of proven GERD that overlap with functional heartburn (164).

Savarino et al. (165) found that the prevalence of DIS was quite low among patients with FH which was comparable to healthy controls when compared with patients with NERD. Also, in the study by Vela et al. (166), the mean intracellular space diameter in patients with GERD was significantly greater than in those with FH and controls, and there was no evidence of esophageal epithelial dilatation in those with FH or controls.

Accordingly, DIS is the most common histopathological feature in the basal epithelial layer of the esophageal mucosa in all patients with GERD, and its presence is considered the most important difference between patients with NERD and those with FH. Since the underlying premise of mucosal impedance is that it is a marker of mucosal integrity, these results suggest that this tool will likely distinguish patients with FH from those with GERD and EoE.

However, likely, a mucosal impedance measurement will not be able to distinguish patients with FH and RH from controls, since recent studies reported that mucosal impedance was lower among patients with GERD, but was similar between those with FH and RH (167). In other words, the presence of normal mucosal impedance in patients with heartburn is suggestive of a functional disorder rather than GERD, which would call for sensory modulation for treatment. Although it has previously been shown that lower baseline impedance was found in RH *versus* FH, it appears that this is inconsistent with the idea that low baseline impedance levels of the esophagus may be associated with high reflux burdens or increased esophageal acid exposure (124). Therefore, further studies are needed.

## Conclusion

In the present study, we conducted a bibliometric analysis of 8,964 publications on GERD research retrieved from the WOSCC of Clarivate Analytics from 2012 to 2022. As a result of the analysis, it was found that current studies in this field focused on the differential diagnosis for GERD, including esophageal motility disorders and functional esophageal disorders, anti-reflux surgery, the reflux-inflammation-Barrett’s cascade, treatment options for GERD in obese patients, and P-CAB, most of which were clinically relevant, and the basic research was insufficient.

Basic research on GERD appears to be lagging, which may be explained by the high focus placed on the role of acid in its pathogenesis for years. Following the degradation of glycoproteins, H^+^ penetrates the esophageal epithelial layer along the intercellular spaces (168). In the presence of DIS, H^+^ can enter the submucosa and interact with sensory afferents, thus leading to symptoms associated with reflux (169). However, an alternative mechanism was proposed by Souza et al. (170), who suggested that bile salts enhanced the stabilization of hypoxia-inducible factor-1a, increasing T lymphocyte-derived chemokines that facilitate the development of esophagitis. This highlights that T lymphocytes mediate the primary mode of injury (171). The GERD pathogenesis, therefore, can be quite complex; it should be viewed as a disorder that goes far beyond acid reflux. Furthermore, the paradox of patients with BE exhibiting few symptoms compared with FH patients having esophageal pain sensitivity indicates the complexity of heartburn generation mechanisms. Research remains to be conducted on the relationship between acid or non-acid reflux and sensory afferents.

From the clinical perspective, as identified in our analysis, there is an increasing trend toward endoscopic- and surgical specialization in GERD research. However, multiple studies that compared GERD patients to non-GERD patients have suggested that GERD and psychological disorders may be interlinked (172). Further, the number of patients with GERD and overlapping functional disorders is increasing, and their clinical and psychological characteristics are comparable to those of conventional functional disorders (173). It is, therefore, necessary for GERD research to involve a collaborative research network consisting of esophagologist, endoscopists, surgeons, endocrinologists, pathologists, and psychiatrists.

Moreover, as mucosal impedance is an emerging technology that has entered the arsenal of the esophageal provider, it has raised awareness that mucosal integrity impairment is present in 68.2–83% of patients with NERD, and 48–100% of those with EE (174). Thus, even though acid has been investigated for many years as a fundamental factor in the pathogenesis of GERD, the availability of other treatments, including hyaluronic acid plus chondroitin sulfate (175), alginates (176) and Rikkushito (177), that are able to strengthen esophageal defenses, has sparked new research in this relevant field and offers opportunities for future research.

## Data availability statement

The original contributions presented in this study are included in the article/Supplementary material, further inquiries can be directed to the corresponding author.

## Author contributions

XT, FW, and BZ led the team and were responsible for all aspects of the project. TZ, WT, XY, XW, YW, and JL substantially contributed to the methods, data acquisition, results, and interpretation. WT, YW, and XY participated in designing and writing the manuscript. TZ, BZ, WT, FW, and XW revised this manuscript critically for important intellectual content. XT gave final approval of the manuscript. All authors contributed to the article and approved the submitted version.
