# Supplementary material for: Trends in gastroesophageal reflux disease research: A bibliometric and visualized study
Source: Front Med (Lausanne). 2022 Sep 29;9:994534. doi: 10.3389/fmed.2022.994534 (PMC9556905; doi:10.3389/fmed.2022.994534)
Supplement: Supplementary file 1 [file Data_Sheet_1.pdf]

Supplementary Material

Search strategy in Science Citation Index Expanded of the Web of Science Core Collection of Clarivate Analytics

((((TI=("gastroesophageal reflux" OR "gastro-esophageal reflux" OR "gastro esophageal reflux" OR "gastric esophageal reflux" OR "gastric oesophageal reflux" OR "gastrooesophageal reflux" OR "gastro-oesophageal reflux" OR "gastro oesophageal reflux" OR "esophageal reflux" OR "oesophageal reflux" OR "acid reflux" OR "reflux esophagitis" OR "reflux oesophagitis" OR "erosive esophagitis" OR "erosive oesophagitis" OR "erosive reflux disease" OR "non-erosive reflux disease" OR "non-erosive esophagitis" OR "non-erosive oesophagitis" OR "endoscopy-negative reflux" OR "endoscopy negative reflux" OR "endoscopy normal reflux" OR "GERD" OR "GORD")) OR AB=("gastroesophageal reflux" OR "gastro-esophageal reflux" OR "gastro esophageal reflux" OR "gastric esophageal reflux" OR "gastric oesophageal reflux" OR "gastrooesophageal reflux" OR "gastro-oesophageal reflux" OR "gastro oesophageal reflux" OR "esophageal reflux" OR "oesophageal reflux" OR "acid reflux" OR "reflux esophagitis" OR "reflux oesophagitis" OR "erosive esophagitis" OR "erosive oesophagitis" OR "erosive reflux disease" OR "non-erosive reflux disease" OR "non-erosive esophagitis" OR "non-erosive oesophagitis" OR "endoscopy-negative reflux" OR "endoscopy negative reflux" OR "endoscopy normal reflux" OR "GERD" OR "GORD")) OR AK=("gastroesophageal reflux" OR "gastro-esophageal reflux" OR "gastro esophageal reflux" OR "gastric esophageal reflux" OR "gastric oesophageal reflux" OR "gastrooesophageal reflux" OR "gastro-oesophageal reflux" OR "gastro oesophageal reflux" OR "esophageal reflux" OR "oesophageal reflux" OR "acid reflux" OR "reflux esophagitis" OR "reflux oesophagitis" OR "erosive esophagitis" OR "erosive oesophagitis" OR "erosive reflux disease" OR "non-erosive reflux disease" OR "non-erosive esophagitis" OR "non-erosive oesophagitis" OR "endoscopy-negative reflux" OR "endoscopy negative reflux" OR "endoscopy normal reflux" OR "GERD" OR "GORD")) AND DT=(Article OR Review)) AND LA=(English)

Timespan: 2012-02-20 to 2022-02-20 (Publication Date)

SUPPLEMENTARY MATERIA

Supplementary Table 1 Top 10 journal and top 10 co-cited journals in GERD research.

Rank	Journal	Count (% of 8,964)	IF	JCR	Rank	Co-cited Journal	Count (% of 150,168)	IF	JCR
1	SURGICAL ENDOSCOPY AND OTHER INTERVENTIONAL TECHNIQUES (the USA)	304 (3.39)	4.584	Q1	1	AMERICAN JOURNAL OF GASTROENTEROLOGY (the USA)	4,953 (3.30)	10.864	Q1

2	OBESITY SURGERY (the USA)	249 (2.78)	4.129	Q1	2	GASTROENTEROLOGY (the USA)	4,467 (2.97)	22.682	Q1
3	DISEASES OF THE ESOPHAGUS (the USA)	234 (2.61)	3.429	Q3	3	GUT (the USA)	4,255 (2.83)	23.059	Q1
4	NEUROGASTROENTEROLOGY AND MOTILITY (England)	219 (2.44)	3.598	Q2	4	ALIMENTARY PHARMACOLOGY & THERAPEUTICS (England)	3,358 (2.24)	8.171	Q1
5	WORLD JOURNAL OF GASTROENTEROLOGY (the USA)	172 (1.92)	5.742	Q2	5	DIGESTIVE DISEASES AND SCIENCES (the USA)	2,832 (1.89)	3.199	Q3
6	DIGESTIVE DISEASES AND SCIENCES (the USA)	153 (1.71)	3.199	Q3	6	CLINICAL GASTROENTEROLOGY AND HEPATOLOGY (the USA)	2,662 (1.77)	11.382	Q1
7	JOURNAL OF NEUROGASTROENTEROLOGY AND MOTILITY (South Korea)	149 (1.66)	4.924	Q1	7	NEW ENGLAND JOURNAL OF MEDICINE (England)	2,435 (1.62)	91.245	Q1
8	JOURNAL OF PEDIATRIC GASTROENTEROLOGY AND NUTRITION (the USA)	129 (1.44)	2.839	Q2	8	WORLD JOURNAL OF GASTROENTEROLOGY (the USA)	2,234 (1.49)	5.742	Q2
9	SURGERY FOR OBESITY AND RELATED DISEASES (the USA)	126 (1.41)	4.734	Q1	9	DISEASES OF THE ESOPHAGUS (the USA)	2,158 (1.44)	3.429	Q3

10	PLOS ONE (the USA)	107 (1.19)	3.240	Q2	10	SURGICAL ENDOSCOPY AND OTHER INTERVENTIONAL TECHNIQUES (the USA)	2,085 (1.39)	4.584	Q1

Supplementary Table 2 Top 10 co-cited references in GERD research.

Rank	Reference	Co-citation	Year
1	Update on the epidemiology of gastro-oesophageal reflux disease: a systematic review	329	2014
2	Guidelines for the diagnosis and management of gastroesophageal reflux disease	314	2013
3	The Chicago Classification of esophageal motility disorders, v3.0	269	2015
4	Modern diagnosis of GERD: the Lyon Consensus	258	2018
5	Eosinophilic esophagitis: updated consensus recommendations for children and adults	149	2011
6	Pediatric gastroesophageal reflux clinical practice guidelines: joint recommendations of the North American Society for Pediatric Gastroenterology, Hepatology, and Nutrition (NASPGHAN) and the European Society for Pediatric Gastroenterology, Hepatology, and Nutrition (ESPGHAN)	132	2009
7	Functional Esophageal Disorders	117	2016
8	Evidence-based clinical practice guidelines for gastroesophageal reflux disease 2015	108	2016
9	Ambulatory reflux monitoring for diagnosis of gastro-esophageal reflux disease: Update of the Porto consensus and recommendations from an international consensus group	106	2017

10	ACG clinical guideline: Evidenced based approach to the diagnosis and management of esophageal eosinophilia and eosinophilic esophagitis (EoE)	99	2013

Supplementary Table 3 Top 5 co-cited references with the highest betweenness centrality in GERD research.

Rank	Reference	Centrality	Year
1	Acid-suppressive medications and risk of oesophageal adenocarcinoma in patients with Barrett's oesophagus: a systematic review and meta-analysis	0.29	2014
2	Long-term proton pump inhibitors and risk of gastric cancer development after treatment for Helicobacter pylori: a population-based study	0.25	2018
3	Systematic review: the effects of long-term proton pump inhibitor use on serum gastrin levels and gastric histology	0.24	2015
4	Does Sleeve Gastrectomy Expose the Distal Esophagus to Severe Reflux?: A Systematic Review and Meta-analysis	0.23	2020
4	Gastroesophageal reflux disease and Barrett's esophagus after laparoscopic sleeve gastrectomy: a possible, underestimated long-term complication	0.23	2017
4	Clinical, Endoscopic, and Histologic Findings at the Distal Esophagus and Stomach Before and Late (10.5 Years) After Laparoscopic Sleeve Gastrectomy: Results of a Prospective Study with 93% Follow-Up	0.23	2019
5	Esophageal baseline impedance levels in patients with pathophysiological characteristics of functional heartburn	0.2	2014

Supplementary Table 4 Overview of co-cited references with the highest betweenness centrality in GERD research.

Reference	Title	Key Findings

(122)	Acid-suppressive medications and risk of oesophageal adenocarcinoma in patients with Barrett's oesophagus: a systematic review and meta-analysis	This meta-analysis of seven observational studies with 2813 patients with BE has found that PPI use is associated with a 71% reduction in the risk of high-grade dysplasia and adenocarcinoma.
(123)	Long-term proton pump inhibitors and risk of gastric cancer development after treatment for Helicobacter pylori: a population-based study	This large-scale, population-based study involving a Hong Kong health database was conducted by Cheung KS et al., who enrolled more than 63,000 patients with *Helicobacter pylori* (*H. pylori*) receiving treatment based on clarithromycin. 153 patients (0.24%) developed gastric cancer during an average follow-up of 7.6 years.
(124)	Systematic review: the effects of long-term proton pump inhibitor use on serum gastrin levels and gastric histology	In this systematic review of the literature that included 16 studies, with a total of 1920 patients, *H. pylori*-positive patients receiving long-term (over 3 years) PPI treatment had a significantly higher risk of developing enterochromaffin-like cell linear/micronodular hyperplasia and corpus atrophy, in comparison to *H. pylori*-negative patients.
(61)	Does Sleeve Gastrectomy Expose the Distal Esophagus to Severe Reflux?: A Systematic Review and Meta-analysis	This meta-analysis including a total of 46 studies involving 10,718 patients evaluating the prevalence of GERD, esophagitis, and BE after LSG, found an overall increase in reflux symptoms in 19%, *de novo* reflux in 23%, esophagitis in 30%, and BE in 8% of patients postoperatively.
(36)	Gastroesophageal reflux disease and Barrett's esophagus after laparoscopic sleeve gastrectomy: a possible, underestimated long-term complication	Genco A et al. studied 110 patients undergoing LSG. The authors performed EGD preoperatively at a mean 58 months of follow-up. It is found that incidence of GERD symptoms, visual analogue scale mean score and PPI intake

		signifcantly increased compared to preoperative values. Endoscopic findings revealed that 17.2% of patients had *de novo* BE. The authors also reported that 26.4% of patients with BE had no GERD symptoms.
(125)	Clinical, Endoscopic, and Histologic Findings at the Distal Esophagus and Stomach Before and Late (10.5 Years) After Laparoscopic Sleeve Gastrectomy: Results of a Prospective Study with 93% Follow-Up	The prospective study conducted by Csendes A et al., involved 104 consecutive patients who underwent sequential clinical, endoscopic, and histologic examinations of the foregut after LSG. Following the preoperative assessment, patients were divided into two groups: patients without reflux (Group 1) and those with reflux (Group 2). The study found that after 10.5 years, *de novo* GERD was detected in 58% of participants in Group 1, whereas remission of symptoms was observed in only 13.6% of participants in Group 2. A large proportion of patients in Group 1 developed hiatal hernia, EE, and dilated cardia.
(126)	Esophageal baseline impedance levels in patients with pathophysiological characteristics of functional heartburn	Martinucci I et al. studied a group of patients with FH, finding lower baseline impedance levels in those who received at least 50% relief after PPI therapy, as compared to healthy volunteers and patients with minimal relief. FH and reflux hypersensitivity may be distinguished based on baseline impedance levels, as those with reflux hypersensitivity generally have lower impedance levels.

Top 25 References with the Strongest Citation Bursts

References	Year	Strength	Begin	End	2012 - 2022
Gyawali CP, 2018, GUT, V67, P1351, DOI 10.1136/gutjnl-2017-314722, DOI	2018	**88.47**	2018	2022	
El-Serag HB, 2014, GUT, V63, P871, DOI 10.1136/gutjnl-2012-304269, DOI	2014	**77.75**	2014	2021	
Katz PO, 2013, AM J GASTROENTEROL, V108, P308, DOI 10.1038/ajg.2012.444, DOI	2013	**71.16**	2013	2021	
Vandenplas Y, 2009, J PEDIATR GASTR NUTR, V49, P498, DOI 10.1097/MPG.0b013e3181b7f563, DOI	2009	**51.29**	2012	2016	
Kahrilas PJ, 2015, NEUROGASTROENT MOTIL, V27, P160, DOI 10.1111/nmo.12477, DOI	2015	**47.94**	2017	2021	
Kahrilas PJ, 2008, GASTROENTEROLOGY, V135, P1392, DOI 10.1053/j.gastro.2008.08.044, DOI	2008	**36.13**	2012	2016	
Liacouras C, 2011, J ALLERGY CLIN IMMUN, V128, P3, DOI 10.1016/j.jaci.2011.02.040, DOI	2011	**31.9**	2012	2016	
ROSEN R, 2018, J PEDIATR GASTR NUTR, V66, P516, DOI 10.1097/MPG.0000000000001889], DOI	2018	**29.26**	2018	2022	
Roman S, 2017, NEUROGASTROENT MOTIL, V29, P0, DOI 10.1111/nmo.13067, DOI	2017	**26.86**	2017	2022	
Eusebi LH, 2018, GUT, V67, P430, DOI 10.1136/gutjnl-2016-313589, DOI	2018	**25.4**	2018	2022	
Peterli R, 2018, JAMA-J AM MED ASSOC, V319, P255, DOI 10.1001/jama.2017.20897, DOI	2018	**24.9**	2018	2022	
Richter JE, 2018, GASTROENTEROLOGY, V154, P267, DOI 10.1053/j.gastro.2017.07.045, DOI	2018	**23.9**	2018	2022	
Genco A, 2017, SURG OBES RELAT DIS, V13, P568, DOI 10.1016/j.soard.2016.11.029, DOI	2017	**23.33**	2017	2022	
Sherman PM, 2009, AM J GASTROENTEROL, V104, P1278, DOI 10.1038/ajg.2009.129, DOI	2009	**22.83**	2012	2016	
Galmiche JP, 2011, JAMA-J AM MED ASSOC, V305, P1969, DOI 10.1001/jama.2011.626, DOI	2011	**22.82**	2012	2016	
Gyawali CP, 2018, GASTROENTEROLOGY, V154, P302, DOI 10.1053/j.gastro.2017.07.049, DOI	2018	**22.08**	2018	2022	
Dellon ES, 2013, AM J GASTROENTEROL, V108, P679, DOI 10.1038/ajg.2013.71, DOI	2013	**21.98**	2013	2021	
Fass R, 2009, GUT, V58, P295, DOI 10.1136/gut.2007.145581, DOI	2009	**21.67**	2012	2016	
Sebastianelli L, 2019, OBES SURG, V29, P1462, DOI 10.1007/s11695-019-03704-y, DOI	2019	**21.1**	2019	2022	
Bredenoord AJ, 2012, NEUROGASTROENT MOTIL, V24, P57, DOI 10.1111/j.1365-2982.2011.01834.x, DOI	2012	**20.93**	2012	2021	
Hvid-Jensen F, 2011, NEW ENGL J MED, V365, P1375, DOI 10.1056/NEJMoa1103042, DOI	2011	**20.92**	2012	2016	
Felsenreich DM, 2017, OBES SURG, V27, P3092, DOI 10.1007/s11695-017-2748-9, DOI	2017	**20.71**	2017	2022	
Souza RF, 2009, GASTROENTEROLOGY, V137, P1776, DOI 10.1053/j.gastro.2009.07.055, DOI	2009	**20.12**	2012	2016	
Iwakiri K, 2016, J GASTROENTEROL, V51, P751, DOI 10.1007/s00535-016-1227-8, DOI	2016	**19.16**	2017	2022	
Wang KK, 2008, AM J GASTROENTEROL, V103, P788, DOI 10.1111/j.1572-0241.2008.01835.x, DOI	2008	**18.77**	2012	2016	

Supplementary Figure 1 Top 25 references with strong citation bursts in GERD research. *Note*: *Year denotes when the article was published. Strength represents the degree of the burst. Blue bars represent the period from 2012 to 2022, while red lines represent the time intervals during which reference bursts occur, i.e. rapid increases in citation counts.*
